# Supplementary material for: Combining Phenylalanine and Leucine Levels Predicts 30-Day Mortality in Critically Ill Patients Better than Traditional Risk Factors with Multicenter Validation
Source: Nutrients. 2023 Jan 27;15(3):649. doi: 10.3390/nu15030649 (PMC9921772; doi:10.3390/nu15030649)
Supplement: Supplementary file 1 [file nutrients-15-00649-s001.zip › nutrients-2175741-supplementary.pdf]

Supplementary Materials:

Table S1. Demographic and laboratory data for the initiation and validation cohorts.

	**Initiation cohort**	**Validation cohort**	
	***n* = 537**	***n* = 139**	***p* Value**
Age (years)	71.5±13.6	70.2±12.6	0.296
Male (%)	323(60.1)	88(63.3)	0.559
APACHE II score	18.8±5.8	19.0±5.7	0.803
SOFA score	7.1±3.3	7.0±3.6	0.717
Co-morbidity			
Diabetes mellitus (%)	256(47.7)	79(56.8)	0.057
Hypertension (%)	350(65.2)	95(68.3)	0.547
Coronary artery disease (%)	231(43.0)	53(38.1)	0.335
Atrial fibrillation (%)	80(14.9)	17(12.2)	0.498
Chronic kidney disease (%)*	156(29.1)	37(31.4)	0.656
Ventilator use (%)	415(77.3)	110(79.1)	0.732
Inotropic agent use (%)	209(38.9)	58(41.7)	0.560
Days in ICU (day)	11.2±7.4	11.5±8.9	0.773
Laboratory data			
Hemoglobin (g/dL)	10.8±2.8	11.0±2.6	0.398
eGFR (ml/min/1.73 m^2)	33.3(12.0-64.9)	36.6(18.9-66.7)	0.188
C-reactive protein (mg/L)	46.8(11.1-104.0)	38.9(15.4-128.5)	0.285
Cholesterol (mg/dL)	132.0±52.9	128.9±49.3	0.530
Triglyceride (mg/dL)	109(81.5-152.0)	111(76-156.5)	0.912
Albumin (g/dL)	3.19±0.62	3.12±0.71	0.351

Data are expressed as the mean ± SD for variables with normal distribution, as the median [interquartile range (IQR)] for variables with skewed distribution, and as a number (percentage) for categorical variables. * Chronic kidney disease is defined as eGFR < 60 ml/min/1.73 m^2. Abbreviations: APACHE II score, Acute Physiology and Chronic Health Evaluation II score; SOFA score, Sequential Organ Failure Assessment score; ICU, intensive care unit; eGFR, estimated glomerular filtration rate.

Table S2. Analysis of the prognostic value of combining phenylalanine and leucine levels to assess 30-day mortality in the initiation cohort (*n* =537).

	**Univariate**		**Multivariable**	
	**HR (95% CI)**	***p* Value**	**HR (95% CI)**	***p* Value**
Phenylalanine				
< 88.5 μM	Reference			
≥ 88.5 μM	3.49 (2.47-4.92)	< 0.001	3.85 (2.67-5.55)	< 0.001
Leucine				
68.9 ~ 165.6 μM	Reference			
> 165.6 μM	1.82 (1.24-2.67)	0.002	1.28 (0.87-1.88)	0.218
< 68.9 μM	1.85 (1.14-3.01)	0.012	2.76 (1.68-4.54)	< 0.001

Abbreviations: HR, hazard ratio; CI, confidence interval.

Table S3. Thirty-day mortality prediction value of different risk levels defined by PLA scores.

**Risk level**	**RR (95% CI)**	***p* Value**
Low	Reference	
Intermediate	2.85 (1.51-5.37)	0.001
High	6.89 (3.46-13.71)	< 0.001
Very-high	19.5 (9.91-38.5)	< 0.001

Abbreviations: PLA score, phenylalanine leucine amino acid score; RR, relative risk; CI, confidence interval.
